# Cell Fate Decisions in the Neural Crest, from Pigment Cell to Neural Development

**DOI:** 10.3390/ijms222413531

**Published:** 2021-12-16

**Authors:** Jonathan H. P. Dawes, Robert N. Kelsh

**Affiliations:** 1 Centre for Networks and Collective Behaviour, University of Bath, Bath BA2 7AY, UK; j.h.p.dawes@bath.ac.uk; 2 Department of Mathematical Sciences, University of Bath, Bath BA2 7AY, UK; 3 Centre for Mathematical Biology, University of Bath, Bath BA2 7AY, UK; 4 Department of Biology & Biochemistry, University of Bath, Bath BA2 7AY, UK

**Keywords:** neural crest cells, peripheral nervous system, sensory neuron, sympathetic neuron, Schwann cell, glia, dorsal root ganglion, fate specification, differentiation, commitment

## Abstract

The neural crest shows an astonishing multipotency, generating multiple neural derivatives, but also pigment cells, skeletogenic and other cell types. The question of how this process is controlled has been the subject of an ongoing debate for more than 35 years. Based upon new observations of zebrafish pigment cell development, we have recently proposed a novel, dynamic model that we believe goes some way to resolving the controversy. Here, we will firstly summarize the traditional models and the conflicts between them, before outlining our novel model. We will also examine our recent dynamic modelling studies, looking at how these reveal behaviors compatible with the biology proposed. We will then outline some of the implications of our model, looking at how it might modify our views of the processes of fate specification, differentiation, and commitment.

## 1. Introduction

**Figure 1:**
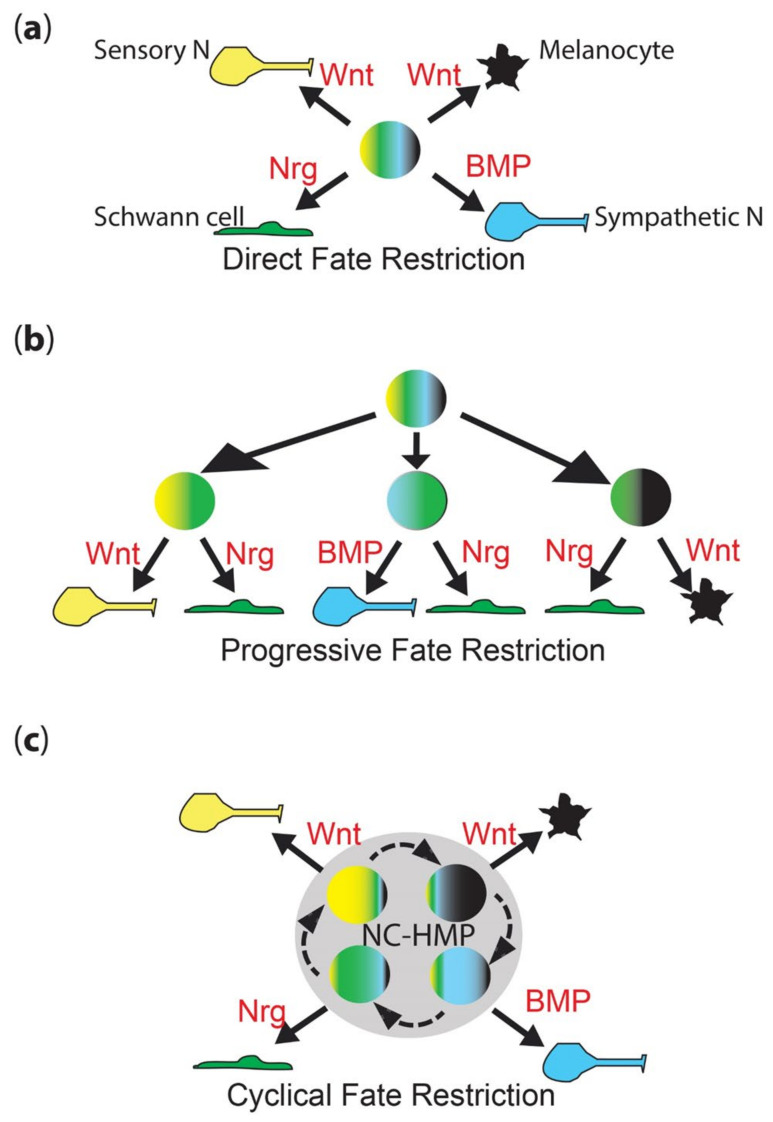
Neural crest fate restriction models. (**a**) Direct fate restriction (DFR). In the DFR model, highly multipotent NCC (rainbow colored) generates each fate directly (solid arrow), under direct influence of environmental signals (shown in red). Work in mammals suggests that Wnt signaling is important for both melanocyte and sensory neuron fate specification, with differences in signal timing being key [27]. (**b**) Progressive fate restriction (PFR). In the PFR model, multipotent NCC rapidly generates a series of intermediate progenitors whose options are restricted. In this schematic, for simplicity, they are shown only as the best-characterized bipotent intermediates (two color), but these models also posit higher potency intermediates as well. The partially restricted (here, bipotent) intermediates then generate individual fates under the influence of environmental signals (red). (**c**) New cyclical fate restriction (CFR) model. In our novel CFR model, NCCs enter a highly multipotent progenitor state (NC-HMP, grey circle), characterized by a highly dynamic transcriptome, which cycles (dashed arrows) through a series of sub-states; note that this state is highly multipotent (rainbow colors), but each sub-state is biased towards an individual fate (broader band of one color); under the influence of environmental signals (red), cells in each sub-state are driven to adopt an individual fate (solid arrow). Note that these simplified schematics focus on only four key fates—melanocyte (black), Schwann cell (green), sensory neuron (yellow) and sympathetic neuron (cyan)—and on key features of each model.

Neural crest cells (NCCs) contribute substantially to the formation of the peripheral nervous system, providing sensory neurons within the dorsal root ganglia, enteric neurons throughout the gut, and sympathetic neurons, as well as the accompanying glial fates, including Schwann cells and satellite glia [1]. However, NCCs also generate a remarkable diversity of other cell types, including cartilage and bone, smooth muscle, adrenal medullary cells, and numerous pigment cells [1]. Early studies showed that many (and presumably all?) early neural crest cells are multipotent, and they are widely considered to at least start off that way [2,3,4,5,6]. A long-standing debate (going back to the 1980s) concerns the mechanism whereby individual fates are chosen. Proponents of the direct fate restriction (DFR) mechanism argue that all fates are directly assigned from fully multipotent cells, whereas others have proposed the progressive fate restriction (PFR) model in which individual fates are selected through a series of intermediate progenitors in which a subset of fate options are retained, but all others are lost (Figure 1a,b) [7,8,9,10,11,12,13,14,15]. These two views have sat uncomfortably together, and have never been well resolved, although PFR has become strongly favored, especially in the context of the peripheral nervous system, where apparently bipotent progenitors for neurons and glial components of sensory and sympathetic ganglia have been characterized.

In contrast, a recent preprint that directly assesses the PFR model for pigment cell development in zebrafish has rejected the PFR hypothesis [16]. This has led us to reassess the debate, and we have recently proposed a novel cyclical fate restriction (CFR) hypothesis (Figure 1c) that can reconcile the conflicting evidence underpinning each of the DFR and PFR models [17]. Here, we will summarize that evidence, and outline the PFR model as an explanation of peripheral neural development. We will then briefly visit the pigment cell field, summarizing the PFR model, and how the new data contradict it. We will outline the CFR model, and briefly consider how it might reconcile the historical models, before summarizing how this might also be consistent with the observations of peripheral nervous system development. We will then discuss a mathematical modelling approach that provides a theoretical underpinning for the CFR model, and will discuss its biological implications and how they might be tested.

## 2. Neural Crest Development

### 2.1. Classical Views of Neural Crest Development

#### 2.1.1. Direct Fate Restriction

Pioneering experiments by Marianne Bronner using iontophoretic dye labelling of neural plate and delaminated NCCs performed on chick embryos observed a mixture of clones contributing to two or more cell types, including peripheral neural, pigment and other fates [13,14,15]. These were interpreted as indicating that neural crest cells might be initially highly multipotent, and then choose fate in accordance with environmental cues in the target destination where differentiation occurs. Thus, cells were considered to migrate in a fully multipotent state. The identification and characterization of neural crest stem cells (NCSCs) by David Anderson’s lab reinforced this view, showing in culture that different environmental cues (growth factors, including BMPs, Neuregulin) drove differentiation of sensory and sympathetic neurons, Schwann cell and smooth muscle fates [18]. Importantly, careful clonal studies demonstrated that the process was instructive, not selective, i.e., the growth factors direct the differentiation of the NCSCs, rather than simply selecting against those that had made the wrong decision through some other mechanism [19,20,21,22,23,24]. Detailed characterization of the process in neuronal development showed the growth factor-dependent transcriptional activation of fate-specific transcription factors was an important initial step in differentiation: thus, for sympathetic neuron development, BMP signaling induces and maintains expression of MASH1 and PHOX2B, whereas for sensory neuron development, Wnt signaling drives Neurogenin1/2 expression [20,21,22,23,24]. Similarly, for Schwann cell fate, both GGF and Notch signaling were crucial, although the key transcription factors affected have remained less clear, with SOX10 important, but with a complex role being involved in neuronal fates too [19,24,25]. Thus, the direct fate restriction (DFR) model posits that fully multipotent cells undergo environmental signal-driven specification directly to individual fates (Figure 1a). When originally proposed, the model was closely linked to the idea that fate specification occurs after migration, consistent with the in vivo observation of high BMP expression in the dorsal aorta adjacent to the site of sympathetic ganglial development [20,26].

#### 2.1.2. Progressive Fate Restriction

Nicole Le Douarin, whose group had observed extensive heterogeneity in clonal composition of primary neural crest cells cultured under conditions conducive to differentiation of diverse cell types, and Jim Weston, noting the heterogeneity of marker expression in premigratory and early migrating neural crest, both suggested an alternative model, progressive fate restriction (PFR; Figure 1b) [7,8,9,10,11,12]. They suggested that initially highly multipotent neural crest cells underwent a series of partial restrictions in their potency, resulting eventually in adoption of a single fate. Again, this was integrated with NCC migration, with the suggestion that the process began prior to or during migration, so that cells arriving at their terminal destination might already be restricted to a subset of fates—and indeed that their migration pathways might be controlled by these restriction decisions. Thus, for example, cells restricted to sympathetic neuron and glial fates (bipotent sympathetic neuroglioblasts) might be guided to the vicinity of the dorsal aorta, forming the nascent sympathetic ganglia; cells that became partially restricted to sensory neuron and glial fate (bipotent sensory neuroglioblasts) would, instead, be directed to the vicinity of the spinal cord. This would be consistent with the striking regularity of NCC migration (but see also more recent evidence arguing that migration in the trunk is rather more random; [28]) and, for melanoblasts on the dorsolateral migration pathway, has been dissected molecularly in chick [29,30,31,32,33,34,35]. The final selection of neuronal versus glial fates would, in each case, result from a local communication process involving both ErbB/neuregulin signaling and Notch-delta lateral inhibition processes [19,25].

### 2.2. Conflicting Observations within Recent Studies

Although widely debated in the late 20th century, the discussion became less prominent subsequently, perhaps because the PFR model became favored. Nevertheless, the issue remained unresolved, and at least one key developmental biology textbook makes reference to both sets of studies, and both ideas, without attempting to resolve them [36]. However, the issue is important, not least because if NCCs work through a DFR mechanism, that is rather distinct from the way we tend to think about development in general. A careful modern clonal fate-mapping study in mice concluded that even migratory NCCs retained multipotency, but in the context of the PFR versus DFR debate that is our focus, it defined multipotency as ‘fated to form at least two cell-types’, a definition that does not distinguish between PFR and DFR [37]. A recent tour de force study of mouse and chick NC development using scRNA-seq generated a molecular representation of the PFR model, with neural development resulting from a series of ‘sequential binary decisions’ with initial coactivation of both programs followed by commitment to one and repression of the other; they describe early segregation of sensory neural progenitors (from which are generated sensory neurons and glia), with other cells then choosing between mesenchymal and autonomic neural fates, and the latter finally choosing between neuronal and glial commitment [38]. Similar conclusions regarding early segregation of neuronally, neuraly and mesenchymally biased progenitors were reached in the context of vagal NC development in chick [39].

#### 2.2.1. Early Fate Specification and Ongoing Multipotency

Examination of key fate-specific markers has expanded the observations of heterogeneity that Weston noted, indicating that many NCCs show evidence of fate specification at premigratory and migratory stages (e.g., [40,41,42,43,44,45,46,47]). Conversely, the identification of NCSCs, and their isolation from multiple locations throughout the body, indicated that at least some individual NCCs retained multipotency during migration and in their post-migratory locations (reviewed in [48]). Some of these cells, most notably those (Melanocyte Stem Cells) giving rise to melanocytes in the adult skin, were initially assumed to be unipotent, but further investigation in mammals showed they had latent multipotency [49,50]. This begs the question of whether these cells are somehow different from the beginning, or is it their final location (the niche) that enables them to retain a cryptic multipotency? Study of pigment cell development in zebrafish embryos has illuminated both these aspects of NCC development.

#### 2.2.2. Zebrafish Pigment Cell Development

Study of NCCs in zebrafish has revealed a strong conservation of derivative cell types and the key genetic factors in their fate specification. In the context of the peripheral nervous system, roles for Neurogenin1, ErbB, Phox2bb, and Sox10 in fate specification of sensory neuron, sympathetic and enteric neuron and glial fates have been shown, paralleling their roles in mammals [51,52,53,54,55,56,57,58]. In the context of the PFR model, bipotent neuroglioblasts have been deduced, with studies of *neurog1* mutants and morphants (embryos generated by injection of morpholinos, and usually showing mosaic knockdown phenotypes) showing that sensory neuroglioblasts adopt glial fates when Neurog1 function inhibited [51].

Particular attention has been focused on the highly visible pigment cell derivatives, partly because zebrafish have melanocytes (often referred to as melanophores) just like mammals, and partly because an abundance of mutants has generated a significant genetic resource for study (e.g., [44,47,59,60,61,62,63,64,65]). Important early work showed that Mitfa, one (of two) zebrafish orthologue of mammalian Mitf, played a pivotal role in melanocyte fate specification from NCCs, with all NC-derived melanocytes being absent in strong loss-of-function mutants, and with expression in the NC being sufficient to rescue *mitfa* mutants [47,66]. Likewise, the role for Sox10 in driving *mitfa* expression has been well demonstrated [67], showing that the role for Sox10 in fate specification of melanocytes is conserved.

**Figure 2:**
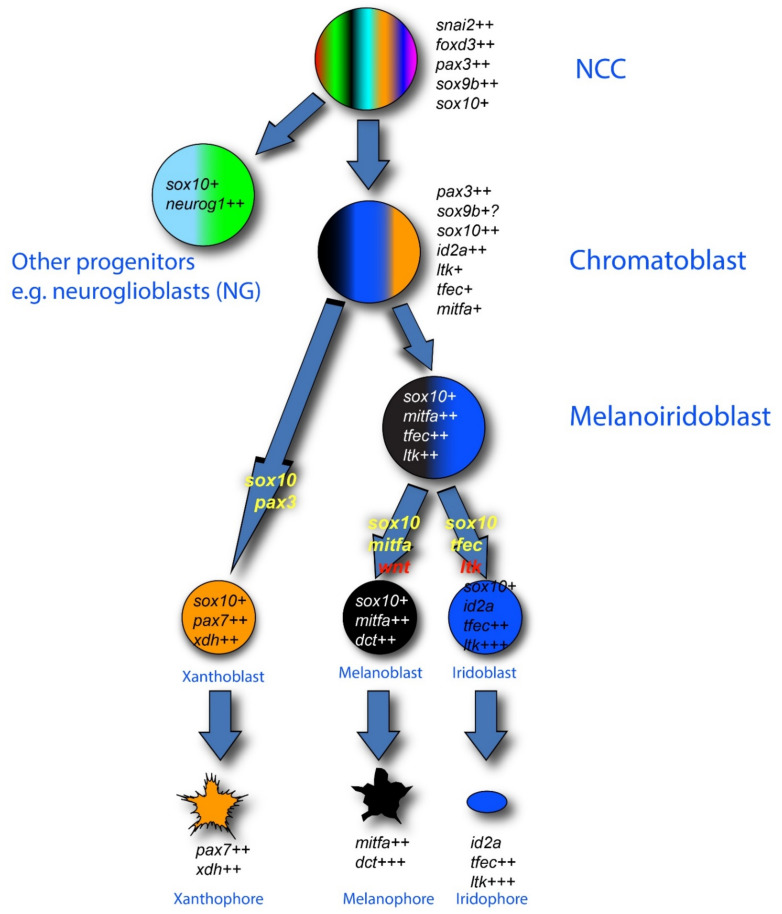
Pigment cell development in zebrafish: conventional PFR model. The three zebrafish pigment cell types (xanthophore (orange), melanocyte (black) and iridophore (blue)) are generated from early neural crest cells (NCCs) by a mechanism involving production of intermediate progenitors that are fate-restricted, respectively, to two or three pigment cell options: melanoiridoblasts (blue, black), generating melanocytes and iridophores only, and chromatoblasts, generating all pigment cell fates (black, blue, orange); other progenitors give rise to other derivatives (e.g., neuroglioblasts (cyan, green) to peripheral ganglia). Key molecular markers as revealed by in situ hybridization are indicated in italics for each proposed cell type; note how recent studies have shown that maintenance and upregulation (rather than de novo transcription) of expression of these markers often characterizes the fate specification and differentiation phases, as indicated here by number of ‘+’ symbols after each gene name. In this diagram, the absence of a marker (e.g., the neuronal transcription factors *neurog1* and *phox2bb*) is significant. Key transcription factor genes (yellow) and environmental signaling molecules (red) driving specific fate choices are indicated on the transition arrows. Note that the development of xanthophores has been less well studied. The mechanisms driving the formation of each intermediate progenitor have not been proposed.

In the context of the fate restriction debate, the real interest in zebrafish stems from the fact that, in fish, there are usually multiple types of pigment cells [68]. In the zebrafish embryo, these consist of melanocytes, but also silver iridophores and yellow xanthophores. Single cell iontophoretic labelling studies have clearly shown that all these pigment cells are NCC derived [55,69,70], whilst genetic studies have identified at least some of the key transcription factors and growth factors and their receptors that are important in their fate specification [41,42,43,44,46,71,72,73,74,75,76]. An influential theory from Joe Bagnara [77] proposed that all pigment cells share a common developmental origin, exclusive to these fates—a classical partially fate restricted progenitor, which we will name a chromatoblast. This idea remained untested, but the emergence of the zebrafish model with its multiple cell fates and genetic tractability, finally opened up the possibility. In addition, a key observation from studies of the conserved role of Mitfa is that the absence of melanocytes is accompanied by a substantial increase in the number of iridophores [47]. This observation, plus similar observations in the context of other pigment cell types in another fish, medaka, were clearly consistent with the idea of bipotent pigment cell progenitors (i.e., here, a melanoiridoblast that forms both melanocytes and iridophores) [78,79,80]. The fit to a PFR model, with the chromatoblast being a more multipotent progenitor of the bipotent melanoiridoblast, was hard to ignore, and led to widespread assumption, by us and by others in the field, that a PFR model would explain zebrafish pigment cell development (Figure 2). Such a view formed the interpretative framework for a number of recent single cell RNA-seq studies, which have identified pigment cell progenitors (putative chromatoblasts) [62,81,82]; only in one case have they been assigned to a melanoiridoblast type, but these cells are from very early larval stages and here there is low-level expression of *pax7b* (a xanthophore lineage marker) too, suggesting they might have higher potency [82].

More directly, our recent attempt to test the PFR model for pigment cell development has refuted the idea. We used sensitive detection of key fate-specific gene expression using Nanostring technology to explore the transcriptome of dissociated zebrafish NCCs throughout the embryonic and very early larval periods, supplemented by both single cell TaqMan assays and sensitive in situ hybridization by RNAscope [16]. Neural crest-derived cells were isolated using fluorescence-activated cell sorting following a transgenic labelling approach. We used a well-characterized *sox10:Cre* driver line [83] to label all neural crest-derived cells after their expression of *sox10*. Nanostring profiling of marker genes was focused on those genes known to have key roles in pigment cell development, especially melanocytes and iridophores, to ensure sensitivity to detection of the proposed intermediates in pigment cell development, but also included selected such markers for other NCC fates; the single cell Taqman assays assessed complementary markers for neuronal fates. Our study revealed the unexpected expression of markers of non-pigment cell fates (including, strikingly, neuronal markers *neurog1* and *phox2b*) overlapping with those of all pigment cell fates; this was even true of control melanocyte and xanthophore cells isolated from 72 hpf larvae. It is currently unclear whether these contrasting observations stem from the increased sensitivity afforded by the Nanostring approach compared to scRNA-seq, or to more extensive disaggregation process leading to cells experiencing a more neutral environment allowing ‘relaxation’ of environmental signal-dependent fate specification, or a mixture of both; however, the co-expression of *phox2b* in many premigratory and a subset of postmigratory cells in glial locations strongly supports the increased sensitivity hypothesis [16], leading us to conclude that these cells are not partially restricted. Hence, we reinterpret the apparent partial restriction seen in ISH studies as partially reflecting technical limitations (most studies only look at one or two markers) and observer bias (where more markers are examined, lower level signals are often dismissed), but also reflecting their environmentally-induced bias towards subsets of fates, rather than the absolute fate restriction. Note that this conclusion would seem equally applicable to neural progenitors as to pigment cell progenitors. Indeed, transgenic fate mapping of cells expressing *leukocyte tyrosine kinase (ltk)*, a marker of premigratory NCCs and of the iridophore lineage, encoding a receptor tyrosine kinase that is crucial for iridophore fate specification and proposed as a marker of putative chromatoblasts in vivo [41], reveals that early *ltk* expressing cells show multipotency, for all pigment cell fates, but strikingly also for neural fates, provides independent support for the conclusion that zebrafish pigment cell progenitors have unexpectedly broad multipotency [16]. Finally, in some cases, we were able to show by RNAscope ISH that post-migratory NCCs showed detectable expression of unexpected combinations of markers, with Schwann cell precursors on the posterior lateral line (sensory) nerve having co-expression of *phox2b* (autonomic neuron) and *ltk* (iridophore) markers [16]. Furthermore, we note that our data provided no evidence for bipotent melanoiridoblasts either, despite the fact that our selected gene set was highly enriched for early melanocyte and iridophore markers. None of these observations are consistent with the PFR model.

A number of recent studies have documented scRNA-seq profiles from zebrafish NC-derived cells [62,81,82]. In general, these have confirmed observations made by standard ISH that premigratory NCCs show early expression of pigment cell markers, indicating early fate specification of these lineages. For example, a recent characterization of 24 hpf trunk NCCs highlighted expression of xanthophore markers [62], confirming old observations of readily-detectable expression in premigratory and migratory trunk NCCs for multiple xanthophore genes; it is worth noting that whilst these cells are likely premigratory, their location adjacent to the epidermis dorsal to the neural tube is also a postmigratory location for xanthophores and melanocytes, so that early differentiation in this region may not be that surprising. However, it has become clear in recent years that these cells, and also migrating NCCs, are often specified to >1 fate, and the scRNA-seq data imply that they may be expressing markers of 2 fates at significant levels [62,81,82]. Detection of such a signature is very much limited by technical sensitivity to low-level expression in these studies, although that is rarely commented upon. The identified cell clusters also show low-level expression (defined as a low proportion of cells showing positive expression) of key markers of other fates too, hinting at the possibility that these cells retain very broad multipotency; thus, the difference between the scRNA-seq and Nanostring data is most likely one of sensitivity of detection of low-level expression, rather than fundamental differences in the biology observed. Indeed, sensitivity of detection appears to be an issue, since the 24 hpf scRNA-seq dataset does not identify *ltk* expression in the putative pigment cell progenitors, despite the fact that this has been repeatedly reported by whole-mount ISH [41,42,43]. This conclusion is reinforced by our observations using an optimized RNAscope approach, where we see distinct, but low level, expression of *phox2bb*, a classic sympathetic/enteric neuron fate specification factor, in unexpected locations, including in numerous glial cells, likely Schwann cell precursors, even in 72 hpf larvae [16]. We would argue that this expression clearly indicates the *potential* of these cells to adopt one of these sympathetic and enteric neuronal fates, regardless of whether this is realized in vivo. (A similar situation is observed for pigment cell markers—although the authors do not comment on this, their quantitative Hybridization Chain Reaction ISH shows wide heterogeneity of expression levels of xanthophore markers *aox5, slc2a15b,* and *gjb8*, with some cells in their figures clearly showing just a handful of mRNAs [62]). We note too that work in mouse and lamprey has recently provided evidence for a contribution of Schwann cell precursors to the enteric neuron population [84,85]; we would predict that the same is likely in zebrafish. Finally, we note how our observations support the growing awareness of the breadth of multipotency of Schwann cell precursors [33,86,87,88,89,90].

#### 2.2.3. Broad Multipotency of Melanocyte Stem Cells

In mammals, hair pigmentation is derived from melanocytes within the hair bulb, with melanosomes being transferred to the keratinocytes that generate the hair shaft (reviewed in [49,91,92,93]). At each hair molt cycle, the hair shaft is regenerated from new keratinocytes derived from keratinocyte stem cells, and melanin is supplied de novo by new melanocytes generated from melanocyte stem cells (MSCs), retained in the hair bulge. In addition, it has been shown in mammals that cells with the stem cell properties of multipotency and self-renewal and capable of forming neurospheres, can be isolated from diverse embryonic (including the original neural crest stem cells (NCSCs) isolated by the Anderson lab from rodent NC) and adult locations including the skin and the gut (reviewed in [48,94,95,96]). Although sharing the properties noted, these NCSCs show differences in their apparent fate biases, reflecting their source location (e.g., [97]). MSCs and NCSCs have been shown to be NC derived; MSCs are probably best thought of as another, perhaps highly specialized, type of NCSC, since they have been shown to be multipotent too [50].

Fish are characterized by distinct embryonic/early larval and adult pigment patterns, with the former widely conserved and likely involved in camouflage and protection of the germline against UV damage, and the latter highly divergent, and important for diverse aspects of adult biology [98,99,100,101,102,103]. Early studies in zebrafish revealed that ablation of pigment cells in the early larva leads to their regeneration, apparently dependent upon a cryptic source of pigment cell precursors [104,105]. Subsequently it was shown that the majority of adult pigment cells are generated de novo during metamorphosis, with the likely exception of an unknown proportion of the adult xanthophores which result from a dedifferentiation-proliferation-differentiation process of the embryonically-derived skin xanthophores [106,107,108,109,110,111]. The source of the de novo pigment cells is hypothesized to be a progenitor cell, likely a stem cell, equivalent to the MSC, and initially named as such. Early studies showed that these cells are NC derived, and that ErbB signaling plays a key role in their being set aside as a quiescent source of pigment cells [106].

However, the identity, number and location of the progenitor cells remain unclear. Pioneering studies indicated that the stem cells are either associated with the peripheral nervous system and/or use the peripheral nerves to deliver their progeny to the skin [112]. A set of cells within the dorsal root ganglia marked with an *mitfa:gfp* transgene became the first well-defined source, and were named MSCs [113], but subsequent work indicates there may well be other such sources associated with other parts of the PNS [114]; indeed it may be that Schwann cell precursors throughout the PNS are (or include) these stem cells [86,112,115,116]. Study of the MSCs soon showed that, in addition to melanocytes, they generate xanthophores and iridophores, but also neurons and glia of the PNS [117]. Thus, they appear to be broadly multipotent, and hence equivalent to the diverse NC-derived neural crest stem cells (NCSCs) identified in mammals, although their strict fulfilment of the stem cell property of self-renewal has not been formally tested. From our perspective here, the key observation is the retention even in the adult of a subset of NC-derived cells that generally sit in a quiescent state, maintained by the local niche, but that retain a clear multipotency when activated, whether at metamorphosis, during regeneration or in tissue maintenance.

### 2.3. Resolving the Conflict?

#### 2.3.1. Cyclical Fate Restriction

The traditional DFR and PFR hypotheses for NC fate acquisition are both partially supported by the evidence, but are distinct and cannot be trivially reconciled. PFR has become the favored mechanism, almost by default, likely because it matches the way that development is usually portrayed, as a series of irreversible, progressive restrictions of available fates, prior to final adoption of a single fate during terminal differentiation. However, the realization that expression of a limited number of key transcription factors can reverse these terminal fates to generate induced pluripotent stem cells (iPSCs; [118]), plus examples of natural fate switching (e.g., [119]), suggest that terminal differentiation is perhaps not quite as terminal as usually portrayed. Furthermore, fate restrictions in PFR are portrayed as a series of bifurcating choices, but is this necessarily the case?

The absence of the well-accepted chromatoblast and melanoiridoblast intermediates in pigment cell development was a major surprise. Although the scRNA-seq studies have named some sort of specified intermediate as a chromatoblast (or similar cell type) [62,81,82], the limitations of the markers available make it unclear what these cells really are; and our Nanostring data argue that they may only be biased towards some of the pigment fates. However, the striking results from *mitfa* and *neurog1* mutant studies, with loss of one cell type (melanocyte or sensory neuron, respectively) being accompanied by an increase in another (iridophore or glia, respectively) [47,51], is not readily accounted for by the DFR model, but demands an explanation.

**Figure 3:**
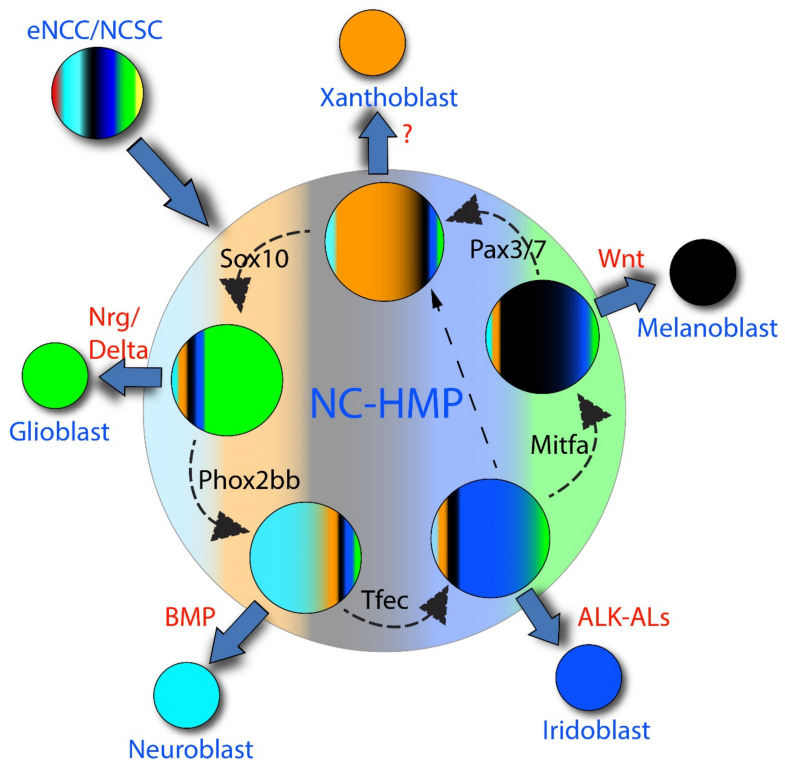
Putative molecular mechanism underpinning CFR. Multipotent neural crest-derived highly multipotent progenitors (NC-HMP) cycles repeatedly through individual sub-states (circles) biased to be receptive to environmental signals driving specific fate choices (indicated by expanded color on each sub-state); we have proposed that this might simply reflect elevated levels of expression of receptors for fate-specific environmental signals (e.g., Ltk for ALK-ALs involved in iridophore specification; BMPRs for BMPs in sympathetic neuron specification). Transition between sub-states (curved, dashed arrows) is proposed to be dependent upon activity of key fate-specific transcription factors (e.g., Tfec for iridophore; Mitfa for melanocyte; Phox2bb and/or MASH1 for sympathetic neurons). Hence, when these genes are mutated, the NC-HMP lingers in the previous sub-state for a longer period (e.g., in *mitfa* mutant, cell lingers in pro-iridophore sub-state), although cells eventually overcome this block using a less favored transition (straight, dashed arrow shown for *mitfa* mutant). Order of transition is predicted based upon mutant phenotypes (e.g., in *mitfa* mutant, iridophore numbers are elevated), but note that order for other fates is rather more hypothetical and subject to further experimental analysis). Key fate specification environmental factors (red) for melanocyte (Wnt), iridophore (ALK-Als), glia (NRG and Notch ligands, e.g., Delta), sympathetic neurons (BMPs) are deduced from zebrafish or mammalian studies; such signal for xanthophore not quite so clear, but Csf1 is a candidate. Note that in this simplified scheme we have assumed just one type of neuron; of course, other neuron types (e.g., sensory neurons) are derived from the NC-HMP.

We have proposed a novel view of NC development, named cyclical fate restriction (CFR) that goes a long way towards reconciling these observations (Figure 1c) [17]. We consider that early NCCs transition to a NC-derived highly multipotent progenitor (NC-HMP) state, characterized by low-level expression of key markers of most or all fates (The ambiguity lies principally in whether or not the skeletogenic fates are excluded). Importantly, we envisage the NC-HMP as displaying a highly dynamic transcriptome, transitioning between states with biases towards one or more states, and cells, at least initially, moving rapidly between such states. At a molecular level, we proposed that the transitioning was an emergent property of the NC-HMP gene regulatory network (GRN), and that it could be influenced by environmental signals, biassing it to certain states, i.e., partially specified cell types (Figure 3). In addition, these biased sub-states would, we proposed, include biases in the sensitivity to environmental fate specification signals, e.g., ALKAL ligands for Ltk and BMPs for BMPRs in the context of cells biased towards iridophore and sympathetic neuron fate, respectively. Although other mechanisms can be readily entertained, this is most simply envisaged as resulting from cyclical expression of the appropriate receptor molecules; we note that our profiling identified two classes of NC-HMPs (named ‘early’ and ‘late’) which are distinguished, in part, by absence or presence of *ltk* expression, respectively, the latter of which conceivably reflects a pro-iridoblast sub-state [16]. The key outcome of these changing sensitivities is that whilst cells retain multipotency they move through states when they are, in turn, biased/fate specified towards different fates, giving the illusion of PFR. In its extreme form, such environmental influences might drive maintenance of the NC-HMP state, as a quiescent NCSC; again, local variations in the environmental signals would be expected to bias the NCSCs to appropriate fates options. The probability of a cell adopting a single fate is influenced by the time spent in the state biased towards that fate, with longer duration increasing the likelihood of differentiation being initiated by prolonged fate specification signals. All these biases would be expected to be lost in a neutral environment, whereupon the underlying broader multipotency would be revealed.

We consider that the switch from non-oscillating early NCC to oscillating NC-HMP happens around or soon after delamination from the dorsal neural tube, so that premigratory NCCs will be NC-HMPs. Consequently, NCCs migrating on each pathway will also be NC-HMPs, but here the specific environmental signals are considered to be instrumental in altering the balance of the different sub-states, so that many (all?) cells display biases towards one or more fates; in the ‘snap-shot’ view of an in situ hybridization experiment, these cells would appear to be fate specified to one or more fates and might be mistaken for PFR intermediate progenitors. Eventually, cells switch from these dynamic states to a committed state, although our evidence suggests this is likely to be surprisingly late in differentiation, since even 72 hpf control melanocytes and iridophores showed evidence of their ability to express key transcription factors of neuronal fates [16].

The dynamic nature of the NC-HMP is a key feature distinguishing CFR from DFR. We have considered a simple DFR model, lacking oscillatory behavior, but with cells retaining basal/poised transcription for some fates whilst displaying elevated levels of genes associated with other fate(s) (i.e., displaying fate specification). This and other dynamic, but non-cyclical, models seem less attractive, because, aside from the difficulty in devising a novel acronym (!), a merely dynamic fate restriction process seems less well suited to explaining the striking mutant phenotypes of fate-specification genes [17]. If we assume that the fate-specific transcription factor function is required for transition to the respective fate-biased state (i.e., Mitfa activity required to transition to a state biased towards melanocyte fate; MASH1 and/or Phox2b required to transition to a sympathetic neuron-biased state), then our model leads to a natural explanation for these mutant phenotypes. The CFR mechanism, with a regular progression from one biased state to the next, suggests that when the progression is blocked, then the preceding fate becomes more likely, since the cell would be expected to remain in the biased state for a longer period; we consider that an alternative, less favored, route to a subsequent biased state will emerge, so that cycling is unlikely to be simply terminated, instead simply ‘slowing down’ in the vicinity of the sub-state upstream of the one blocked by the mutation. Thus, unlike the DFR model, the CFR model provides a satisfying explanation for a key piece of the evidence underpinning the PFR model.

#### 2.3.2. Dynamic Modelling of Fate Restriction Reproduces Key Features Consistent with Cyclical Fate Restriction

In the course of formulating the CFR hypothesis, we asked whether a molecular mechanism could be envisaged that would exhibit the cyclical behavior required; our modelling investigations generated some striking observations that show interesting parallels to the biology [120]. Of course, it is important to remember that, like most mathematical models, the system is highly simplified and thus must be interpreted cautiously; nevertheless, the outputs of the modelling showed several striking features that at least provide some support for the possibility of the behavior the CFR model requires. Amongst several ideas we considered, a modified version of the classic repressilator circuit [121] shows great promise, as we now explain.

**Figure 4:**
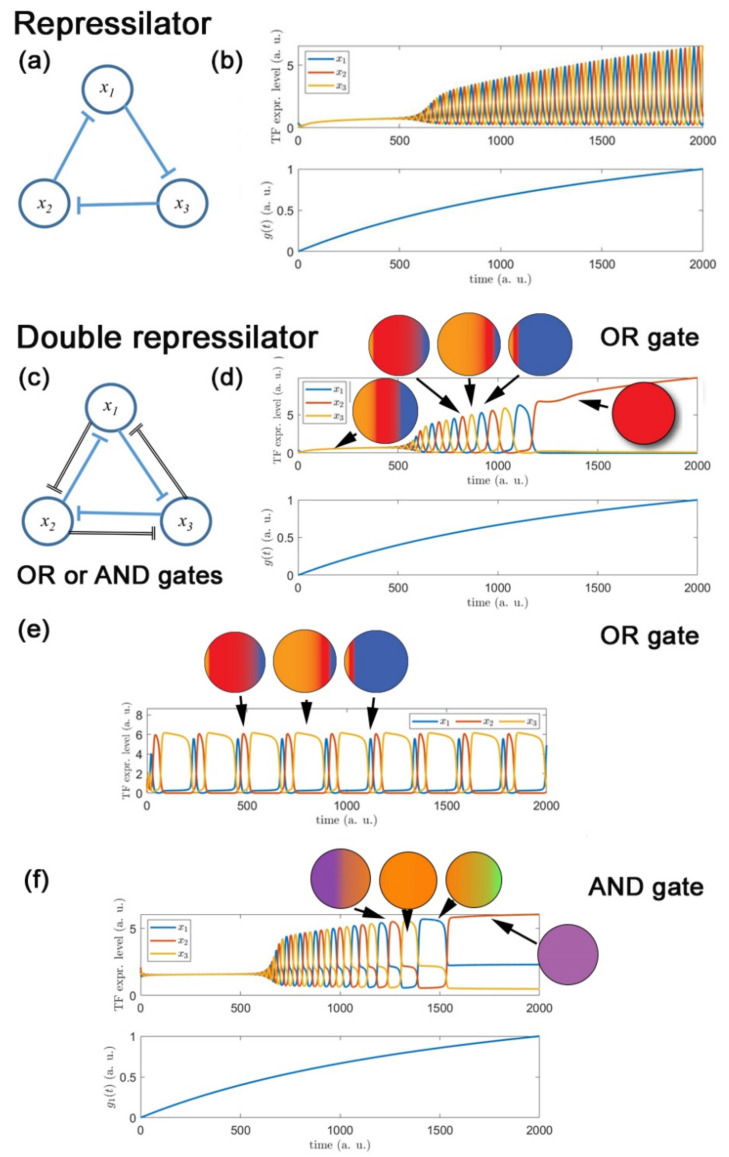
Simple genetic regulatory networks of mutual inhibition and their temporal response to a steadily increasing strength of inhibitory coupling *g(t)*: (**a**,**b**) Cyclic repression between three genes (*x_1_–x_3_*) generates oscillatory transcription factor (TF) expression levels as shown by the repressilator circuit; (**c,d**) Two superimposed sets of mutual inhibition between three genes, with equal inhibitory strengths and using OR gate (i.e., repression occurs so long as one of TFs is expressed), allows for non-oscillatory, multipotent state (panel (**d**), left), oscillatory expression (giving cells that in a snap-shot view appear fate specified, but which retain multipotency (central region of panel (**d**), with three distinct biased sub-states depicted diagrammatically) and also for equilibrium states in which one TF is expressed at much higher levels than the other two, i.e., differentiation (panel (**d**), right). (**e**) If inhibitory strengths between TFs are asymmetric, this creates oscillation between sub-states, but with temporal bias to one, i.e., biased sub-state favoring specific fate; here (in contrast to the other panels) shown for constant coupling strength, i.e., *g(t) = const*. (**f**) Where inhibitions between TFs use AND gate (i.e., both required for the repression), oscillatory sub-states and resultant differentiation states combine expression of 2 TFs, consistent with combinatorial fate determination.

The original repressilator GRN, illustrated in Figure 4a, consists of three transcription factors linked in a loop of inhibitory influences—each transcription factor inhibits the expression of another (downstream) transcription factor, and then is itself inhibited by the remaining transcription factor which lies upstream of it. Theoretical modeling and experimental reconstruction of the repressilator in *E. coli* demonstrates that this circuitry readily and robustly generates temporally oscillating expression of each transcription factor over a wide range of parameter values Figure 4b [121]. The only other natural response mode for the repressilator GRN is an equilibrium state in which all three TFs are expressed at constant, and approximately equal, levels. Such an equilibrium, with all three TFs being co-expressed, we interpret as the cell being in a highly multipotent state. This equilibrium response mode occurs when the strengths of the inhibitory influences are not sufficiently strong to overcome the balancing background processes of production and degradation. Since the repressilator does not contain any other equilibrium states in which levels of TFs differ significantly from each other, we conclude that the standard repressilator GRN is not able to represent both highly multipotent and fate-specialized states which we require in order to be able to model the emergence of multiple fates.

However, it turns out that a simple and natural modification of the standard repressilator GRN does allow the emergence of multiple fates, and moreover provides a robust pathway between highly multipotent and fate-specialized states [120]. The key modification of the standard repressilator is to incorporate mutual repression between every pair of TFs, with potentially unequal strengths in the repressions that act in one cyclic direction compared to those that act in the opposite direction around the circuit, as illustrated in Figure 4c. This ‘double-repressilator’ circuit has three natural modes of response: an equilibrium in which all TF levels are equal (Figure 4d, left), oscillatory expression levels (Figure 4d, center), and novel equilibria in which one TF is expressed at a much higher level than the other two (Figure 4d, right). As reported in Farjami et al. (2021), the two different kinds of equilibrium state appear at low and high levels of parameters that describe the overall level of interaction between the three genes, with the oscillatory regime providing a natural intermediate regime that mediates between them. This has a natural interpretation in terms of dynamical evolution from a highly multipotent state, through an oscillatory state in which the cell makes repeated transitions between transient “sub-states” in which it is biased in favor of a specific fate, but has not yet made a fate selection, through to stable acquisition of a single fate.

Where the cross-repression strengths are set equal, oscillatory behavior shows symmetrical cycling of each transcription factor, so that sub-states biased to each of the derivative fates are equally weighted (Figure 4d), and as the external signal increases, the specific fate stably adopted is determined by the transcription factor expression levels at the time when the external signal exceeds the oscillatory boundary [120]. Our modeling also revealed that exit from the oscillatory regime to a stable state (fate) can also be driven by a second external signaling factor that is fate-specific, e.g., Ltk signaling for iridophores.

In our modelling, we observed that adjustments in the strength of the mutual inhibition between transcription factors (as might be expected under the influence of environmental signals) readily shifts the balance, so that one sub-state is favored during the oscillatory phase; thus, these environmental influences generate a biased progenitor state (Figure 4e).

A subtlety in the modelling of the double repressilator is the need to consider whether the two inhibitory signals act separately on each target or only in concert [120]. We term these the OR gate and AND gate versions, respectively; the scenario described above refers to the OR gate configuration in which high expression levels of either of the other two TFs serve to inhibit expression of the third one.

The difference in behavior between the OR gate and AND gate configurations (in which only high levels of both other TFs provide inhibition) is that in the latter we see oscillation between sub-states in which two of the three transcription factors are co-expressed (Figure 4f) [120]; this would appear to match the biological observations of co-expression of multiple specification factors (i.e., partially specified cells), which give the impression of partially restricted cell types. This is an intriguing finding, in that the instantaneous snap-shots of genetic profiles provided by single-cell analysis, or by in situ hybridization studies using 2–3 key markers, could reflect merely the variety of these sub-states that are available at any one time point, rather than any partially fate-restricted near-equilibrium intermediate states.

Finally, expansion of the repression system to incorporate four or five or more key transcription factors to represent elevated multipotency of the progenitor allows further elaboration of the combinatorial expression of transcription factors during the oscillatory and final stably differentiated states [120]. We note that, at least in the simplest models of this type, for even numbers of transcription factors cross-repression must act also between non-neighboring transcription factors in the network, whereas for odd numbers of genes, the oscillatory behavior is retained in the case that the repression arrows are restricted to immediate upstream and downstream neighbors.

### 2.4. Implications and Further Work

#### 2.4.1. Fate Specification to Multiple Fates Simultaneously

Although in some ways CFR seems to combine key aspects of DFR and PFR, it is distinctive primarily in the dynamic nature of the transcriptome during the differentiation process. This means that while influenced by local environmental signals cells can be held in states where they are specified to two or more fates at any time, and thus in the static snap-shot view of ISH they can be (over-)interpreted as partially fate-restricted. However, the clear prediction is that they will, even if only transiently, cycle on through another sub-state. This can be readily enhanced by removing those local environmental signals, hence the key prediction that under conditions whereby cellular dissociation is prolonged, the apparent fate specification will collapse, with the GRN returning to a more ‘basal’ stem cell state.

Although the concept of development as a series of bifurcations is widespread, it is not intuitively obvious why fate choices, even in a PFR context, need always be so. Indeed, a recent study of cell fate assignment during mouse gastrulation provides evidence in another developmental context for the kind of multiple fate choice decision-making that we are proposing in the neural crest [122]. Our double-repressilator models can readily be expanded to larger numbers of transcription factors, representing increased numbers of potential fates, and oscillatory behavior is readily seen for all models in which there are an odd number of transcription factors [120]. Intriguingly, oscillatory expression in these models includes options with more than one transcription factor gene being expressed simultaneously, recalling the partially specified cell states observed by RNAscope in vivo and scRNA-seq. In the generic double-repressilator models that we have explored so far, the co-expressed transcription factor genes are characterized by being *non*-adjacent in the interaction network. We expect that careful quantitation of the co-expressed genes across all cell types would help illuminate the order of transcription factors in the circuit and the inhibitory interactions between them.

For simplicity of modelling, we used the simplification of the master regulator concept, i.e., that a single key transcription factor determines a specific fate. However, recent work favoring a more distributed control of pigment cell differentiation [43,123], with multiple transcription factors acting combinatorially, can readily be incorporated within our model; indeed, it emerges from modelling of double-repressilator models with AND gates for the repression activities [120]. The distributed control view is looking more realistic [122]; indeed, combinatorial action of transcription factors within neuron fate determination is a well-established idea, with PHOX2B and NEUROG1 being only key parts of the profile driving sympathetic/enteric and sensory neuron development, and is equally readily incorporated within the CFR model. A rapidly growing body of work, focused on chick, mouse, zebrafish and frog, has used both classical genetic and modern ‘omics approaches to define the GRN of NC induction, fate choice and differentiation [32,39,41,42,43,56,123,124,125,126,127,128,129,130,131,132,133,134,135,136,137,138,139,140,141,142,143,144,145,146,147]. For example, two key studies in chick identified a hierarchy of changing regulatory landscapes and transcriptional profiles over time [39,130], which we would interpret as the impact on NC-HMPs of the environmental signals that they have encountered, reflecting both their current environment, but also the history of such exposures. A key task now is to sift that data to identify key players and to examine their GRN relationships to assess whether they might form regulatory structures consistent with the proposed generation of oscillatory behavior, and then to test directly for such behavior in vivo. For example, it is interesting in the light of our double-repressilator model to note that cross-regulatory mutual repression is a common feature of the combinatorial transcription factor codes characterizing different fate choices (e.g., [32,87,130]).

#### 2.4.2. Cyclical Gene Expression

Fundamental to our model is the cyclical expression of some key genes. Specifically, we predict oscillating expression of key transcription factors (likely to include Mitfa, Neurog1, and Phox2bb; although we note that oscillating *activity* of the transcription factors, rather than strictly oscillating mRNA and/or protein expression levels per se, would be an alternative equally consistent with our model) and, as explained above, fate-specification receptors (including *frizzled, ltk, notch)*. The proponents of the PFR model emphasized the heterogeneous nature of marker expression in the early NCCs, and we propose that some of this may result from such oscillations. We consider that *ltk* expression is a prime candidate for investigation here in the context of iridophore development. Moreover, oscillations of Notch expression have been observed in a CNS context, and indeed have been ascribed important roles in controlling neuronal differentiation [148]. Might similar mechanisms be important in fate specification in the NC?

An interesting observation from the modelling was that oscillatory behavior only emerged within certain values of a control parameter representing an environmental signal, with cells in a single stable state at parameter values below (and for the simple repressilator, also above) this critical range. This immediately brings to mind, and could perhaps explain, the observation of a notable delay in the onset of *mitfa* expression in premigratory NCCs. Experimental evidence in zebrafish makes clear that both Sox10 and Wnt signaling are required for *mitfa* transcription [55,67,149,150,151]. Expression of *sox10* initiates around 11 hpf [55], whereas *mitfa* begins at around 18 hpf [47]. The timing of Wnt signaling is harder to define, partly because the specific ligands and receptors involved in *mitfa* expression in zebrafish are not well defined, so conceivably delayed Wnt might explain the delay in *mitfa* expression. Could it be that the *levels* of Wnt signaling lie below the values needed for oscillation, and it is only then that *mitfa* expression becomes possible? Transgenic reporters for Wnt signaling now allow us to investigate this, since we would predict that the levels of signaling would change coincident with the onset of *mitfa* expression.

#### 2.4.3. The Time Scale of Oscillations

What is less clear is the timescale of such oscillations, although we imagine that, for them to be meaningful in terms of a cell’s response the period in zebrafish is likely to be of the order of minutes rather than seconds.

#### 2.4.4. When Does Commitment Occur?

Our Nanostring data showed several unexpected features [16]. One was the widespread expression of multiple fate-specific transcription factors even at late stages, e.g., 72 hpf, when it is assumed that cells have committed to specific fates. Sensitive in situ hybridization using RNAscope identified widespread expression of *phox2bb* in cells of the PNS and with a morphology suggesting they were glial cells, most likely Schwann cell progenitors. This begs the question of when these cells become committed to a Schwann cell fate? Even more surprising was that even control 72 hpf melanocytes and iridophores, isolated by their physical properties (density differences after differentiation) showed profiles after disaggregation that, whilst clearly of differentiated cell types with robust expression of multiple differentiation markers, also showed expression of other key fate-specific transcription factors, including *phox2bb, mitfa,* and *tfec* and fate specification receptors (*ltk*). It is currently unclear to what extent this reflects very low-level expression of these markers in differentiating cells, or whether it reflects a partial relaxation of the GRN following cell isolation; either way it suggests that their commitment to pigment cell fates at that stage may not have been complete.

Another way of viewing this is to consider that the early (and maybe later?) differentiated states are under active maintenance by local signals. Under this view, we can consider that SCPs, which have been shown to be multipotent NCSCs [86,88,152,153,154], and other NCSCs are NC-HMPs that have a histological and molecular phenotype driven by the local niche (e.g., for SCP, including the neuronal axon; [87]); consistent with this, the transcriptomic signature of SCPs and NCCs in mice are remarkably similar [38]. Release from that niche (e.g., when SCPs disperse from the axons; [86]) results in a change in the GRN state so that the NC-HMP resumes its fate sub-state cycling mode; this might be a key aspect of stem cell activation. Furthermore, the CFR model helps us understand how the specific local environments of such activated NCSCs will constrain the likely derivative fates in a spatially-appropriate manner.

#### 2.4.5. Environmental Signals and NC-HMP Fate

Our model indicates the enormous significance of environmental signals encountered by NCCs prior to, during and after migration in instructing NCC fate. Examination of transcriptional profiles of NCCs by scRNA-seq and in situ hybridization only indirectly indicate the signals received by these cells. Whilst some of these signals and their likely regions of action have been identified (e.g., BMPs in sympathetic neuron fate specification; [21,26]), our knowledge is incomplete and, in particular, we have little knowledge of the quantitative and spatial and temporal changes in NCC exposure to each signal during development. The use of signaling pathway reporter lines (e.g., [155,156,157,158]) should provide significant input here. In addition, localized manipulation of such signals, examining the changing transcriptional state of adjacent NC-HMPS, will be a revealing test of the reversibility of apparent ‘fate restriction’ events.

## 3. Conclusions

In summary, a long-standing conflict in the understanding of how NCCs become assigned to their diverse fates remains an important, but recently somewhat neglected, debate. We believe that the current models cannot be reconciled, but that a novel dynamic cyclical fate restriction model provides a stimulating framework within which to reevaluate old and new data. Here, and elsewhere [17], we have proposed how this model might be tested, by noting its distinctive features and their implications. Experimental and ongoing theoretical investigations will enable their assessment in the coming years, although technical advances in live reporting of mRNA expression may be required to achieve the sensitivity predicted to be necessary.

## Data Availability

Not applicable.
